# Immunochromatographic Strip Based on Tetrahedral DNA Immunoprobe for the Detection of Aflatoxin B_1_ in Rice Bran Oil

**DOI:** 10.3390/foods13152410

**Published:** 2024-07-30

**Authors:** Lin Xu, Wenli Qu, Xiaotong Hao, Min Fang, Qing Yang, Yuzhi Li, Zhiyong Gong, Peiwu Li

**Affiliations:** 1 College of Food Science and Engineering, Wuhan Polytechnic University, Wuhan 430023, China; qu2294760111@163.com (W.Q.); xiaotonghao0303@163.com (X.H.); fangmin0227@126.com (M.F.); qingyang@whu.edu.cn (Q.Y.); yuzhili_226@163.com (Y.L.); gongwhqg@163.com (Z.G.); 2 Key Laboratory for Deep Processing of Major Grain and Oil, Ministry of Education, Wuhan 430023, China; 3 Hubei Key Laboratory for Processing and Transformation of Agricultural Products, Wuhan 430023, China; 4 Key Laboratory of Detection Technology of Focus Chemical Hazards in Animal-Derived Food for State Market Regulation, Hubei Provincial Institute for Food Supervision and Test, Wuhan 430075, China; 5 National Reference Laboratory for Agricultural Testing (Biotoxin), Laboratory of Quality and Safety Risk Assessment for Oilseed Products (Wuhan), Key Laboratory of Detection for Mycotoxins, Quality Inspection and Test Center for Oilseed Products, Ministry of Agriculture and Rural Affairs, Oil Crops Research Institute, Chinese Academy of Agricultural Sciences, Wuhan 430062, China

**Keywords:** tetrahedral DNA nanostructure, immunoprobe, immunochromatographic assay, aflatoxin B_1_

## Abstract

Aflatoxin B_1_ (AFB_1_), a widespread contaminant in food and feeds, poses a threat to the health of animals and humans. Consequently, it is significant to develop a rapid, precise and highly sensitive analytical method for the detection of AFB_1_. Herein, we developed an immunochromatographic strip (ICS) based on a tetrahedral DNA (TDN) immunoprobe for AFB_1_ determination in rice bran oil. Three sizes of TDN immunoprobes (AuNP-TDN_13bp_-mAb, AuNP-TDN_17bp_-mAb, AuNP-TDN_26bp_-mAb) were constructed, and the performance of these three immunoprobes, including the effective antibody labeling density and immunoaffinity, was measured and compared with that of the immunoprobe (AuNP-mAb) developed using the physical adsorption method. Subsequently, the optimal TDN immunoprobe, namely AuNP-TDN_13bp_-mAb, was selected to prepare the immunochromatographic strip (ICS) for the qualitative and quantitative detection of AFB_1_ in rice bran oil. The visual limits of detection (vLODs) of the ICS based on AuNP-TDN_13bp_-mAb and AuNP-mAb were 0.2 ng/mL and 2 ng/mL, with scanning quantitative limits (sLOQs) of 0.13 ng/mL and 1.4 ng/mL, respectively. The ICS demonstrated a wide linear range from 0.02 ng/mL to 0.5 ng/mL, with good specificity, accuracy, precision, repeatability, and stability. Moreover, a high consistency was observed between the constructed ICS and ultra-high-performance liquid chromatography (UPLC) in the quantification of AFB_1_. The results indicated that the introduction of TDN was beneficial for promoting efficient antibody labeling, protecting the bioactivity of immunoprobes, and increasing the sensitivity of detection, which would provide new perspectives for the achievement of the highly sensitive detection of mycotoxins.

## 1. Introduction

Aflatoxins are toxic secondary metabolites mainly produced by *Aspergillus flavus* and *Aspergillus parasiticus* [1,2]. Among them, aflatoxin B_1_ (AFB_1_) is classified as a Group I carcinogen by the International Agency for Research on Cancer (IARC) due to its high toxicity [3]. Excessive and prolonged exposure to AFB_1_ can result in teratogenic, mutagenic, and carcinogenic diseases, posing a significant threat to animals and human beings [4]. Furthermore, AFB_1_ is widely present in agricultural products and feeds, whose contamination can occur in any procedure from farm to table [5]. Rice bran oil, as a prevalent and nutritious vegetable oil, is rich in gluten, tocopherol, phytosterol, and other nutrients and is extracted from rice bran, which is susceptible to being contaminated with aflatoxins [6,7,8,9]. Due to its intense toxicity and widespread distribution, many countries and regions have set maximum levels of AFB_1_. Hence, it is crucial to establish a rapid, efficient, and sensitive method for the detection of AFB_1_ to ensure global food safety [10,11].

At present, one of the typical analytical approaches available for AFB_1_ detection focuses on instrumental analysis, mainly including liquid chromatography–mass spectrometry (LC-MS) and high-performance liquid chromatography (HPLC) [12,13,14]. Despite their reliability and accuracy, these methods often require expensive equipment and technical personnel for precise quantification. In contrast, enzyme-linked immunosorbent assay (ELISA)-based detection methods have gained wide application due to their low-cost and straightforward operation [15,16]. Nevertheless, the time-consuming incubation and washing procedures restrict their practical application. Therefore, it is imperative to establish a method for the rapid on-site detection of AFB_1_ [17].

Compared with the aforementioned methods, the immunochromatographic strip (ICS) method, which is rapid, convenient, simple, and affordable, has been widely used in the detection of AFB_1_. However, the sensitivity of conventional ICSs based on colloidal gold nanoparticles (AuNPs) is relatively low [18]. Researchers have exerted numerous efforts to improve the sensitivity of AuNP-based ICSs. Novel nanoparticles are receiving increased attention due to their large specific surface area and unique physical properties, which are capable of capturing more antibodies or amplifying the readout signal for improvements in their sensitivity [19,20,21]. However, the orientation of the antibody on the solid-phase carrier is of significance in establishing a highly sensitive detection method since the ideal orientation of an antibody could fully expose the antigen-binding sites, giving rise to the intense recognition between antibodies and targets. To address this issue, tetrahedral DNA nanostructures (TDNs) were incorporated into the assembly process of the immune probe.

A TDN is a type of three-dimensional (3D) pyramid structure formed through the self-assembly of four complementary single-strand DNA (ssDNA) by annealing. Owing to its advantages such as simple synthesis, structural rigidity, and high programmability, it has been extensively employed in the field of biosensing [22,23,24]. Lin et al. [25] developed a biosensor using TDN, which can precisely tune the anchoring density, by designing different sizes of TDNs [26,27,28,29,30]. Currently, TDNs are mostly used in electrochemical detection, primarily on two-dimensional electrodes. Inspired by these advancements, we endeavored to attach TDNs to 3D colloidal gold particles to accurately control the antibody density with the aim of significantly enhancing the target accessibility and the signal sensitivity [31,32,33,34,35].

In this study, three immunoprobes were constructed based on TDNs with different diameters. The distinctive characteristic of TDN was employed to improve the labeling density of the effective antibodies, protecting the antigen-binding fragment (Fab) from sheltering due to the disordered orientation of antibodies. Subsequently, the immunoprobe with the best performance was selected for the development of ICSs for the highly sensitive detection of AFB_1_ in rice bran oil. It is worth noting that the introduction of TDN greatly facilitates the effective labeling of antibodies, which protects the bioactivity of the antibody, reduces the dosage of the antibody, and makes cost savings possible. More importantly, the site-directed immobilization of antibodies is conducive to the specific recognition of the antibody and target, which has the potential to be extended to the establishment of highly sensitive detection methods for other contaminants.

## 2. Materials and Methods

### 2.1. Reagents and Materials

AFB_1_, ochratoxin A (OTA), zearalenone (ZEN), toxin-2 (T-2), and fumonisin B_1_ (FB_1_) were purchased from Alta Technology Co., Ltd. (Tianjin, China). Anti-AFB_1_ monoclonal antibody (AFB_1_-mAb) and AFB_1_ antigen (AFB_1_-BSA) were purchased from Wuxi Determine Biotechnology Co., Ltd. (Wuxi, China). The DNA sequences shown in Table S1 were synthesized and purified by Biological Engineering Co., Ltd. (Shanghai, China). Colloidal gold (AuNP) was obtained from Nanjing XFNANO Materials Tech Co., Ltd. (Nanjing, China). Polyclonal antibody (pAb) was supplied by Shandong Lvdu Biotechnique Co., Ltd. (Binzhou, China). Horse radish peroxidase (HRP)-labeled polyclonal antibody (IgG-HRP) was purchased from White Shark Biotechnology Co., Ltd. (Hefei, China). The HNO_3_, H_2_SO_4_, HCl, Na_2_EDTA, KOH, K_2_CO_3_, sucrose, Tween-20 (analytical grade), and bovine serum albumin (BSA) were obtained from Sigma-Aldrich Co., Ltd. (Urbana, IL, USA). Tris-(2-carboxyethyl) phosphine hydrochloride (TCEP, 98%, CAS No.: 51805-45-9) was obtained from Macklin Biochemical Co., Ltd. (Shanghai, China). Dimethyl sulfoxide (DMSO), Azido-PEG4-NHS Ester (AEPS), and MgCl_2_ were purchased from Sinopharm Group Protection Chemical Reagent Co., Ltd. (Shanghai, China). The PAGE gel kit and gel red nucleic acid dye were provided from Beyotime Biotechnology Co., Ltd. (Shanghai, China). Phosphate-buffered saline (PBS, 10 mmol/L, pH 7.4), borate buffer solution (BBS, 0.05 mol/L, pH 8.0), TM buffer (20 mol/L Tris, 50 mol/L MgCl_2_, pH 8.0), and carbonate buffer solution (CBS, 0.05 mol/L, pH 9.6) were provided by Cytiva Co., Ltd. (Shanghai, China); bran oil was commercially purchased from the local supermarket. The Sartorius CN140 nitrocellulose (NC) membrane, sample pads, polyvinyl chloride (PVC), and absorbent pads were obtained from Jieyi Biotechnology Co., Ltd. (Shanghai, China).

### 2.2. Equipment

To prepare the strip, an XYZ 3035 Dispensing Platform and a ZQ 2002 Guillotine Cutter were provided by Gold Label Biotechnology Co., Ltd. (Shanghai, China). An Enspire multimode plate reader was obtained from PerkinElmer Instruments Co., Ltd. (Waltham, MA, USA). A Python Quartz Crystal Microbalance (QCM) was provided by Boinst Technology Co., Ltd. (Beijing, China). Dynamic light scattering (DLS) equipment was obtained from Brookhaven Instruments Co., Ltd. (Holtsville, NY, USA). A Tanon-5200 multi-automatic gel imaging analysis system was supplied by Tanon Technology Co., Ltd. (Shanghai, China). Polymerase chain reaction (PCR) kits were provided by Brookhaven Instruments Co., Ltd. (Holtsville, NY, USA). An ultraviolet–visible (UV–Vis) spectroscope was obtained by Brookhaven Instruments Co., Ltd. (Holtsville, NY, USA).

### 2.3. Self-Assembly of TDNs

TDNs were synthesized according to the previous literature [36]. In brief, four DNA strands (A, B, C, D) were mixed in TM buffer to a final concentration of 1 μmol/L and reacted with TCEP. An equal amount of DNA was denatured at 95 °C for 10 min, followed by rapid annealing at 4 °C for 30 min to produce TDNs (TDN_13bp_, TDN_17bp_, TDN_26bp_). Then, the synthesized TDNs were characterized by poly acrylamide gel electrophoresis (PAGE) and the fluorescence resonance energy transfer (FRET).

### 2.4. Construction and Characterization of the Immunoprobes

**Figure 1:**
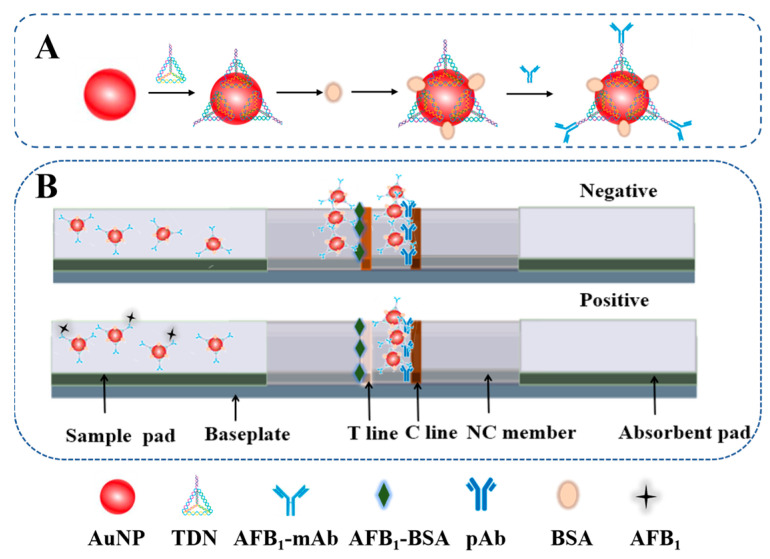
A schematic diagram of immunochromatographic strip (ICS) for detecting AFB_1_. (**A**) Construction of AuNP-TDNs-mAb; (**B**) fabrication of ICS.

The immunoprobes, namely AuNP-TDNs-mAb conjugates, were prepared as follows (Figure 1A): Optimal amounts of TDN_13bp_, TDN_17bp_, and TDN_26bp_ were dropped into 1 mL of AuNP and incubated at room temperature (RT) for at least 16 h to obtain the AuNP-TDNs (AuNP-TDN_13bp_, AuNP-TDN_17bp_, and AuNP-TDN_26bp_) conjugates. Upon stirring for 2 h, 50 µL of 10% BSA was added to seal the excess binding sites on the AuNP surfaces. Subsequently, the AuNP-TDNs conjugates were purified by centrifugation and resuspended in 1 mL of PBS. Then, 70 µL of the click chemical reaction liquid (0.12 g/mL AEPS) was added and allowed to react at RT for 3 h. Then, the optimal amount of mAb was added and incubated at RT for 2 h. Eventually, these three probes were purified by centrifugation and resuspended in 100 µL of running buffer (PBS containing 0.1% Tween-20 and 1% sucrose) for further use. Additionally, the immune probe (AuNP-mAb) based on physical adsorption labeling was prepared according to a previous report with minor modifications [37]. To verify the successful construction of these immunoprobes, ultraviolet–visible absorption spectrum (UV–Vis), dynamic light scattering (DLS), a quartz crystal microbalance (QCM), and the zeta potential were utilized to characterize these three immunoprobes (AuNP-TDN_13bp_-mAb, AuNP-TDN_17bp_-mAb, AuNP-TDN_26bp_-mAb).

### 2.5. Estimation of the Labeling Density of Effective Antibody

The optimal amount of TDN was added to AuNP to obtain AuNP-TDN conjugates, followed by the addition of excess mAb, which were then incubated for 2 h at RT to acquire AuNP-TDN-mAb complexes. The coupling density of the antibody was calculated by determining the antibody in the supernatant before and after reaction using Coomassie bright blue. Thereafter, excess antigen was added and incubated along with the antibodies of the immunoprobe for 2 h at 37 °C, followed by the measurement of the unbound antigen via Coomassie bright blue. Ultimately, the labeling density of the effective antibody was estimated by measuring the consumption of the antigen captured by the antibody of the AuNP-TDN-mAb probe. The coupling density of the antibody and the labeling density of the effective antibody of AuNP-mAb were also estimated based on the above.

### 2.6. Investigation of the Immunoaffinity of the Immunoprobes

The immunoaffinity of these three AuNP-TNDs-mAb and AuNP-mAb immunoprobes was detected using ELISA. Briefly, a 96-well microplate was coated with AFB_1_-BSA (1 μg/mL, 100 μL/well) in CBS buffer and incubated overnight at 4 °C, followed by six washes with PBST (PBS containing 0.05% Tween-20). After washing, 200 μL of 1% BSA was added to each well, which was incubated for 2 h at 37 °C. Subsequently, a series of concentrations (0.01, 0.02, 0.05, 0.1, 0.2, 0.4, and 0.5 mg/mL) of each immunoprobe were added successively and incubated for another 1 h at 37 °C. Then, 100 μL of 3.2 μg/mL IgG-HRP was added and incubated for 1 h at 37 °C, followed by the addition of TMB (100 μL) as substrate. After 15 min of incubation, 100 μL of H_2_SO_4_ was added to terminate the reaction, and the absorbance of the mixture was measured immediately at 450 nm. The Hill equation curves of these four immunoprobes (AuNP-mAb, AuNP-TDN_13bp_-mAb, AuNP-TDN_17bp_-mAb, and AuNP-TDN_26bp_-mAb) were fitted, for which the dissociation equilibrium constant (K_d_) values were utilized to evaluate the immunoaffinity [38].

### 2.7. Sample Pretreatment

Firstly, 5 g of rice bran oil sample was transferred into a 50 mL centrifuge tube and mixed with 20 mL of 70% methanol, followed by 20 min of vortexing. After centrifugation at 6000 rpm for 10 min, the supernatant was filtered through a 0.22 μm membrane for further use.

### 2.8. Fabrication of the ICS

The AuNP-TDN_13bp_-mAb immunoprobe with the optimal performance was selected for the preparation of ICS (Figure 1B) and was compared to the ICS based on AuNP-mAb. The ICS consists of four parts, namely a polyvinyl chloride (PVC) baseplate, a nitrocellulose (NC) membrane, a sample pad, and an absorbent pad. The sample pads were blocked with PBS containing 0.05% (*v*/*v*) Tween-20, 1% BSA, and 2.5% (*m*/*v*) sucrose, followed by drying overnight at 37 °C. Then, 0.5 mg/mL of pAb (0.5 mg/mL) and the optimal amount of AFB_1_-BSA were sprayed onto the NC membrane as the control (C) line and test (T) line at a rate of 0.8 μL/cm. The distance between the C and T lines was set to 8 mm. Next, the sample and absorbent pad were sequentially attached to both sides of the PVC baseplate with a 2 mm overlap and then divided into strips with a width of 4.5 mm.

The dosage of AuNP-TDN_13bp_-mAb and the spray concentrations of AFB_1_-BSA on T line were optimized based on the chessboard method. The spray concentration of AFB_1_-BSA was set to 0.3, 0.4, and 0.5 mg/mL, while the dosage of the AuNP-TDN_13bp_-mAb probe was set to 6, 7, 8, 9, and 10 µL, respectively. Under optimal conditions, ICSs were used to detect AFB_1_ standard solutions with concentrations of 0 and 10 ng/mL. To achieve the best detection performance, both the color development of the T line and the detection sensitivity were taken into consideration.

### 2.9. Qualitative and Quantitative Detection of AFB_1_ by ICSs

The appropriate amount of AuNP-TDN_13bp_-mAb probe was thoroughly mixed with the sample extraction of rice bran oil and fully blended to obtain a 100 μL solution. Then, the test strip was inserted into the mixture and incubated at 37 °C for 15 min for the qualitative detection of AFB_1_ by observing the color of the T and C lines. The visual limit of detection (vLOD) was defined as the concentration at which the minimum T line was significantly shallower than that of the negative control. The cut-off value was the concentration at which the T line completely vanished.

Meanwhile, the grayscale values of the C (GSc) and T (GS_T_) lines were recorded with a scanner and quantitatively analyzed using ImageJ 2.12 software . The GS_T_/GS_C_ values of the negative control and spiked samples were defined as GS_0_ and GS, respectively. A series of spiked rice bran oil with final AFB_1_ concentrations of 0.02, 0.05, 0.1, 0.2, 0.3, 0.4, 0.5, 0.6, 1, and 2 ng/mL were analyzed to establish the standard curve for the quantitative analysis. Particularly, the scanning limit of quantification (sLOQ) was defined as the concentration of AFB_1_ corresponding to the value of the GS_0_ mean plus the 10-fold standard deviation (SD) of the negative samples. Additionally, the spiked rice bran oil samples were simultaneously detected by this proposed immunosensor and ultra-high-performance liquid chromatography (UPLC), and then the *t*-test was conducted for significance analysis [39].

## 3. Results

### 3.1. Characterization of the TDNs

**Figure 2:**
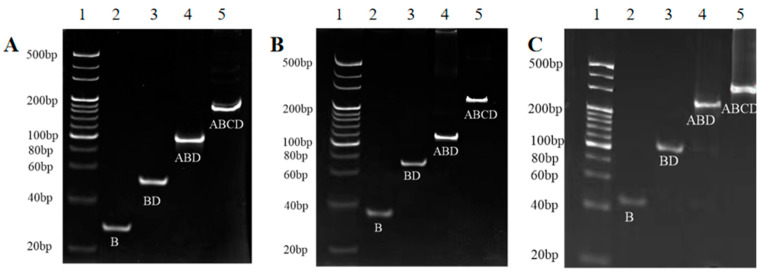
Characterization of tetrahedral DNA nanostructure (TDN) via gel electrophoresis. (**A**) TDN_13bp_; (**B**) TDN_17bp_; (**C**) TDN_26bp_. Lane 1: DNA marker; lane 2: B; lane 3: BD; lane 4: ABD; lane 5: ABCD.

**Figure 3:**
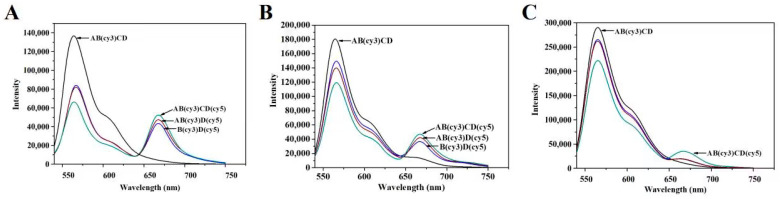
Characterization of tetrahedral DNA nanostructures (TDNs) via fluorescence resonance energy transfer (FRET). (**A**) TDN_13bp_; (**B**) TDN_17bp_; (**C**) TDN_26bp_.

The successful preparation of TDNs (TDN_13bp_, TDN_17bp_, and TDN_26bp_) was crucial for the subsequent construction of immunoprobes. Hence, 10% native PAGE was employed to characterize the stepwise synthesis of the TDNs with diverse sizes. As depicted in Figure 2A–C, the single B chain possessing a low molecular weight demonstrated the fastest mobility. With the synthesis of the TDNs, the spatial complexity as well as the molecular weight gradually increased, hindering the movement of the DNA strands. Consequently, the migration velocity of the BD and ABD hybrid chains was lower than that of the B chain. When the A, B, C, and D chains were incubated together, the slowest mobility emerged due to the formation of a TDN. To further certify the integrity of the TDNs, the B and D strands were, respectively, modified with fluorophore and quencher to investigate the FRET efficiency. As shown in Figure 3A–C, with the synthesis of TDNs, the FRET enhanced since the distance between the quencher cy5 and fluorophore cy3 decreased. In conclusion, TDNs with diverse sizes were successfully synthesized.

### 3.2. Characterization of Immunoprobes

**Figure 4:**
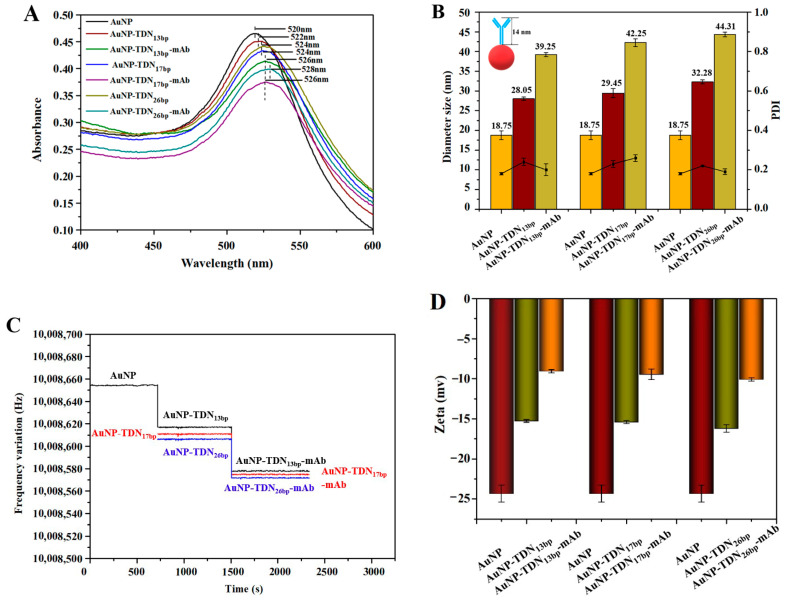
Characterization of AuNP-TDN_s_-mAb immunoprobes. (**A**) Ultraviolet–visible absorption spectrum (UV–vis); (**B**) dynamic light scattering (DLS); (**C**) quartz crystal microbalance (QCM); (**D**) zeta potential.

UV–vis absorption spectroscopy was carried out to confirm the successful preparation of these three AuNP-TDNs-mAb composites. In Figure 4A, a characteristic absorption peak of AuNP is presented at 520 nm. With the successive modification of the TDNs and mAb on the AuNP surfaces, the wavelength of the maximum adsorption displayed obvious redshifts, giving rise to the successful synthesis of AuNP-TDNs and AuNP-TDNs-mAb. In the DLS characterization (Figure 4B), the diameters of AuNP-TDN_13bp_-mAb, AuNP-TDN_17bp_-mAb, and AuNP-TDN_26bp_-mAb increased from 18.75 nm to 28.05, 29.45, 32.28 nm, and then to 39.25, 42.25, and 44.31 nm, respectively. The estimated values were quite close to the theoretical values both for the TND diameter and for mAb with the ideal orientation. Conversely, the QCM frequency of these three probes decreased due to the increased mass after binding with the TDN and mAb (Figure 4C). Furthermore, the zeta potential showed a significant increase from −24 to about −9 mv for AuNP-TDNs-mAb (Figure 4D), demonstrating the successful synthesis of immunoprobes.

### 3.3. Determination of the Labeling Density of the Effective Antibody

Upon the addition of the appropriate TDNs (Figure S1) and excessive mAb, AuNP-TDNs-mAb was produced. To evaluate the performance of these three immunoprobes, the antibody coupling density and the effective antibody density were determined and compared to those of AuNP-mAb. As shown in Table 1, the antibody coupling densities of AuNP-TDN_13bp_-mAb, AuNP-TDN_17bp_-mAb, AuNP-TDN_26bp_-mAb, and AuNP-mAb were 19, 16, 8, and 31 per AuNP, while the effective antibody densities of these four immunoprobes were 18, 16, 8, and 10 per AuNP, respectively, with effective labeling rates of 94.7%, 100%, 100%, and 32.3%, correspondingly. Although the AuNP-TDNs-mAb captured fewer antibodies, they exhibited high proportion of effective antibodies. This implies that the antibodies of AuNP-TDNs-mAb are beneficial to the extensive exposure of Fab, which are prone to ideal head-on orientation to improve the effective labeling of antibodies and further enhance the recognition between antibody and analyte.

### 3.4. Investigation of the Immunoaffinity

**Figure 5:**
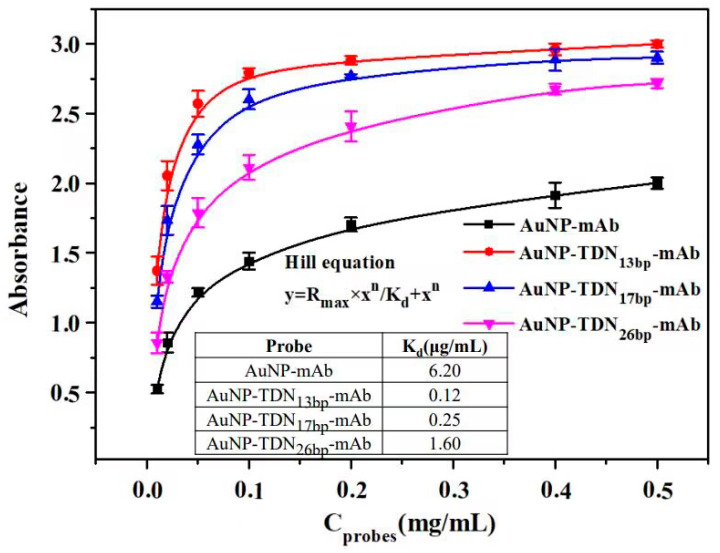
Hill equation curves and K_d_ values of probes. K_d_: dissociation equilibrium constant.

The Hill equation was fitted and utilized to evaluate the immunoaffinity of the immunoprobes via the K_d_ value, which are negatively correlated. As illustrated in Figure 5, the K_d_ values of the AuNP-mAb, AuNP-TDN_13bp_-mAb, AuNP-TDN_17bp_-mAb, and AuNP-TDN_26bp_-mAb immune probes were 6.2, 0.12, 0.25, and 1.6, respectively, indicating that the AuNP-TDNs-mAb immunoprobes exhibited stronger binding than AuNP-mAb. The introduction of TDN facilitated the specific recognition between antibodies and antigens. Considering the immunoaffinity and the effective labeling density of the antibody, the AuNP-TDN_13bp_-mAb immunoprobe, due to its excellent performance, was selected for the subsequent ICS experiments.

### 3.5. AFB_1_ Analysis of the Proposed ICS

**Figure 6:**
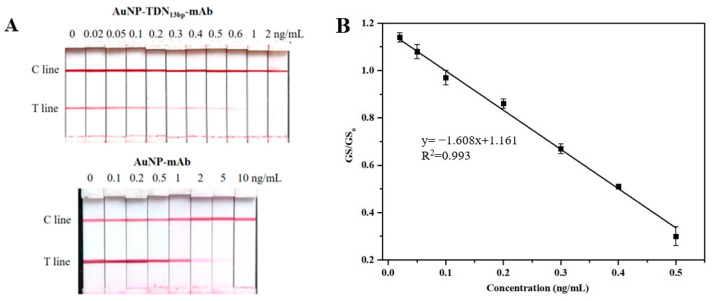
(**A**) Sensitivity analysis of the immunochromatographic strips (ICSs) based on AuNP-TDN_13bp_-mAb and AuNP-mAb; (**B**) calibration curve of the ICS based on AuNP-TDN_13bp_-mAb.

A total of 9 μL of AuNP-TDN_13bp_-mAb was mixed with 91 μL of AFB_1_-spiked sample extract. The mixture was analyzed by means of the proposed ICS, on which 0.3 mg/mL AFB_1_-BSA was sprayed on the T line. Under the optimal conditions, the vLOD of the ICS was 0.2 ng/mL with a cut-off value of 1 ng/mL (Figure 6A), which represents a significant improvement compared to that of the AuNP-mAb-based strip (vLOD, 2 ng/mL; cut-off value, 10 ng/mL). Moreover, the GS values of the C and T lines were measured by ImageJ software for a quantitative analysis. As shown in Figure 6B, the linear range of the quantification for the fabricated ICS was from 0.02 to 0.5 ng/mL. Correspondingly, the equation of the standard curve was y = −1.608x + 1.161, with a correlation coefficient (R^2^) of 0.993. The sLOQ of the ICS based on the AuNP-TDN_13bp_-mAb was 0.13 ng/mL, which is an increase of one order of magnitude compared to that of the ICS based on the AuNP-mAb probe (1.4 ng/mL).

### 3.6. Assessment of the ICS

**Figure 7:**
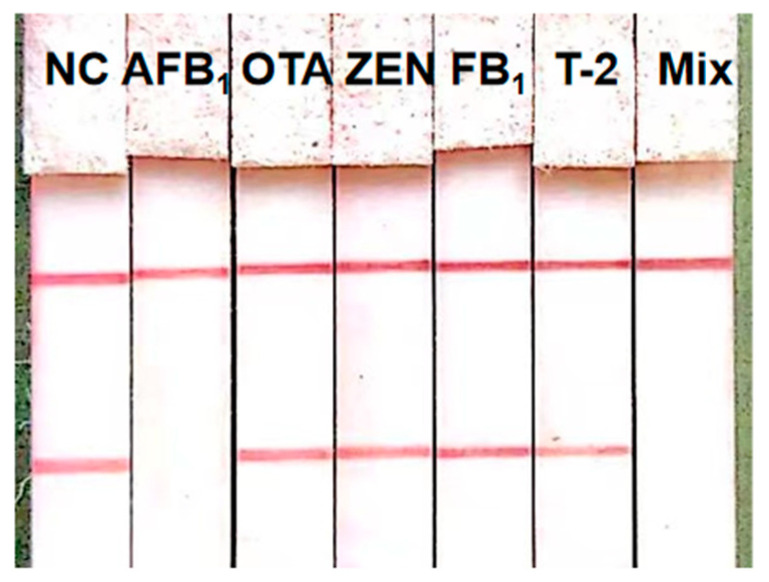
Specificity of the immunochromatographic strip (ICS).

The specificity was evaluated by simultaneously detecting target mycotoxin AFB_1_ and four common non-target mycotoxins, namely OTA, ZEN, FB_1_, and T-2. As indicated in Figure 7, AFB_1_ could be specifically identified by the ICS. More importantly, the existence of all other mycotoxins, whether together or in isolation, did not have an impact on the response to AFB_1_. Only an evident red band at the T line was observed for the detection of AFB_1_, indicating the high selectivity of this proposed method.

In the repeatability experiment (Table 2), ICSs from the same batch were used to detect AFB_1_ at concentrations of 0.05, 0.1, 0.3, 1, and 2 ng/mL. Only one false negative was observed, perhaps because the concentration of AFB_1_ was close to the vLOD, so was prone to being overlooked with the naked eye.

Meanwhile, the intra-assay and inter-assay precision of this proposed ICS was evaluated via recovery experiments. The results (Table 3) shows that the intra-assay and inter-assay recoveries ranged from 85.0% to 98.2% with a coefficient of variation (CV) varying from 5.23% to 7.47%, indicating acceptable accuracy and precision for the rapid quantitative screening of AFB_1_.

Furthermore, this developed ICS was compared with other immunochromatographic assays for the detection of AFB_1_. As shown in Table 4, the proposed ICS demonstrated a lower _V_LOD, good specificity, accuracy, and acceptable repeatability. Particularly, when compared with the random labeling of antibodies, the sensitivity was improved by the introduction of TDN, which could promote the specific recognition of the antibody and the target aflatoxin.

### 3.7. Method Verification Using UPLC

Rice bran oil samples spiked with different concentrations of AFB_1_ were used to evaluate the practicability of the ICS. As shown in Table 5, the results for the ICS and UPLC had no significant difference according to the *t*-test statistical method (|t| = 0.079, t_α_/2 = 2.132, |t| < t_α_/2). All these results illustrate that the ICS is applicable for the detection of AFB_1_ in practical samples.

## 4. Conclusions

In this study, three novel immunoprobes based on TDNs with diverse diameters were constructed and applied for the fabrication of highly sensitive ICSs for the detection of AFB_1_ in rice bran oil. With the introduction of TDNs, the effective antibody labeling density was extremely improved compared to that of the immunoprobe prepared via the adsorption labeling method, whose orientation is beneficial for fully exposing the Fab sites on the mAb. Considering the immunoaffinity and the effective labeling density of the antibody, AuNP-TDN_13bp_-mAb, which showed excellent performance, was selected to fabricate the ICS for the qualification and quantification of AFB_1_. Under the optimal conditions, the vLOD was 0.2 ng/mL, and the sLOQ was 0.13 ng/mL, with a linear range of 0.02–0.5 ng/mL. Thanks to the introduction of the TDN, the sensitivity of this proposed strip increased by one order of magnitude compared to that of the ICS based on AuNP-mAb.

In addition, the performance of the developed ICS was assessed by determining spiked samples, and the ICS displayed good specificity, repeatability, accuracy, and precision. Additionally, the results of the spiked recovery experiments were consistent with those of the UPLC, which can fulfill the requirements for the national standard limit of AFB_1_ in rice bran oil. Therefore, the utilization of TDN was conducive to the enhancement in the specific recognition of antibodies, leading to an improvement in sensitivity, which provides a theoretical basis for further developing a highly sensitive detection method for AFB_1_.

## Figures and Tables

**Table 1:** The labeling density of the antibody.

Immune Probe	Antibody Coupling Density (mAb/AuNP)	Effective Antibody Density (Effective mAb/AuNP)	Proportion of Effective Antibodies (%)
AuNP-TDN_13bp_-mAb	19	18	94.7
AuNP-TDN_17bp_-mAb	16	16	100
AuNP-TDN_26bp_-mAb	8	8	100
AuNP-mAb	31	10	32.3

**Table 2:** Analysis of repeatability.

Spiked (ng/mL)	ICS Visual Observation Results (*n* = 20)	
	Detection Results	Abnormality Rate%
0	+ ^a^	0
0.05	+	0
0.1	+	5
0.3	± ^b^	0
1	− ^c^	0
2	−	0

^a^ Shows color; ^b^ color lightened obviously; ^c^ no color; abnormality rate % = (fn/20) × 100, fn is the number of repeats of abnormal color development of the detection line.

**Table 3:** The precision of the ICS method for AFB_1_ detection in spiked rice bran oil samples.

AFB_1_ (ng/mL)	Intra-Assay (*n* = 6)			Inter-Assay (*n* = 6)		
	Mean ± SD (ng/mL)	Recovery (%)	CV (%)	Mean ± SD (ng/mL)	Recovery (%)	CV (%)
0.1	0.095 ± 0.03	95.0	6.17	0.093 ± 0.03	93.0	7.47
0.3	0.267 ± 0.06	89.0	8.03	0.255 ± 0.05	85.0	7.12
0.5	0.491 ± 0.02	98.2	5.23	0.480 ± 0.05	96.0	6.78

SD: standard deviation; CV: variable coefficient.

**Table 4:** Comparison of the detection performance to other immunochromatographic assays.

Probe	_V_LOD (ng/mL)	Specificity (Interfering Mycotoxins)	Accuracy	Repeatability (Abnormality Rate)	Ref.
AuNP-mAb	2	AFB_2_/AFG_1_/AFG_2_	cd-ELISA	/	[40]
AuNP-mAb	1	AFB_2_/AFG_1_/AFG_2_	ic-ELISA HPLC	10% (ic-ELISA)	[41]
AuNP-mAb	0.5	AFB_2_/AFG_1_/AFG_2_/ OTA/Citrinin/ Patulin/ZEN/T-2	HPLC	/	[42]
AuNP-mAb	0.5	/	ELISA	/	[43]
GNPs-mAb	0.5	AFB_2_/AFG_1_/AFG_2_	/	0%	[44]
GNP-mAb	0.5	ZEN/T-2/FB_1_/ DON/OTA	/	/	[45]
AuNP-TDN-mAb	0.2	OTA/ZEN/FB_1_/T-2	UPLC	5%	This study

Accuracy: The detection results are in good accordance with the method used for comparison shown in the table; GNPs: gold nanoparticles; AFB_2_: aflatoxin B_2_; AFG_1_: aflatoxin G_1_; AFG_2_: aflatoxin G_2_; OTA: ochratoxin A; ZEN: zearalenone; DON: deoxynivalenol; FB_1_: fumonisin B_1_; cd-ELISA: competitive direct enzyme-linked immunosorbent assay; ic-ELISA: indirect competitive ELISA; HPLC: high-performance liquid chromatography; UPLC: ultra-high-performance liquid chromatography.

**Table 5:** Comparison of the analytical results between the developed ICS and UPLC (*n* = 3).

Sample	Spiked (ng/mL)	Mean (ng/mL) ± SD (ng/mL)	
		ICS	UPLC
Rice bran oil	0.05	0.053 ± 0.036	0.055 ± 0.018
	0.1	0.108 ± 0.022	0.094 ± 0.019
	0.3	0.307 ± 0.051	0.325 ± 0.034
	0.5	0.511 ± 0.016	0.469 ± 0.051

SD: standard deviation.

## Data Availability

The original contributions presented in the study are included in the article/Supplementary Material, further inquiries can be directed to the corresponding authors.

## Supplementary Materials

The following supporting information can be downloaded at: https://www.mdpi.com/article/10.3390/foods13152410/s1, Table S1: DNA sequences used in this study; Figure S1: Optimization of the amount of TDNs.
