# Supplementary material for: Immunochromatographic Strip Based on Tetrahedral DNA Immunoprobe for the Detection of Aflatoxin B1 in Rice Bran Oil
Source: Foods. 2024 Jul 30;13(15):2410. doi: 10.3390/foods13152410 (PMC11311855; doi:10.3390/foods13152410)
Supplement: Supplementary file 1 [file foods-13-02410-s001.zip › foods-3096457-supplementary.pdf]

Supporting Information

Table S1 DNA sequences used in this study

Nucleic acid	Sequence (5’-3’)	nt
A13-L	ACACTACGTCAGAACAGCTTGCATCACTGGTCACCAGAGTA**GTATCCAGTGGCTCA**	56
B13	ACGAGCGAGTTGATGTGATGCAAGCTGAATGCGAGGGTCCT	41
cy3-B13	**cy3-**TCAACTCGCTCGTAACTACACTGTGCAATACTCTGGTGACC	41
C13	TCAACTCGCTCGTAACTACACTGTGCAATACTCTGGTGACC	41
D13	TCTGACGTAGTGTATGCACAGTGTAGTAAGGACCCTCGCAT	41
D13-cy5	TCTGACGTAGTGTATGCACAGTGTAGTAAGGACCCTCGCAT**-cy5**	41
A17-L	ACATTCCTAAGTCTGAAACATTACAGCTTGCTACACGAGAAGAGCCGCCATAGTA<br>**GTATCCAGTGGCTCA**	70
B17	TATCACCAGGCAGTTGACAGTGTAGCAAGCTGTAATAGATGCGAGGGTCCAATAC	55
cy3-B17	**cy3-**TATCACCAGGCAGTTGACAGTGTAGCAAGCTGTAATAGATGCGAGGGTCCAATAC	55
C17	TCAACTGCCTGGTGATAAACGACACTACGTGGGAATCTACTATGGCGGCTCTTC	55
D17	TTCAGACTTAGGAATGTGCTTCCCACGTAGTGTCGTTTGTATTGGACCCTCGCAT	55
D17-cy5	TTCAGACTTAGGAATGTGCTTCCCACGTAGTGTCGTTTGTATTGGACCCTCGCAT**-cy5**	55
A26-L	GCCTGGAGATACATGCACATTACGGCTTTCCCTATTAGAAGGTCTCAGGTGCGCGTTT<br>CGGTAAGTAGACGGGACCAGTTCGCC**GTATCCAGTGGCTCA**	99
B26	CGCGCACCTGAGACCTTCTAATAGGGTTTGCGACAGTCGTTCAACTAGAATGCCCTTT<br>GGGCTGTTCCGGGTGTGGCTCGTCGG	84
cy3-B26	**cy3-**CGCGCACCTGAGACCTTCTAATAGGGTTTGCGACAGTCGTTCAACTAGAATGCCC<br>TTTGGGCTGTTCCGGGTGTGGCTCGTCGG	84
C26	GGCCGAGGACTCCTGCTCCGCTGCGGTTTGGCGAACTGGTCCCGTCTACTTACCG<br>TTTCCGACGAGCCACACCCGGAACAGCCC	84
D26	GCCGTAATGTGCATGTATCTCCAGGCTTTCCGCAGCGGAGCAGGAGTCCTCGGCCTTT<br>GGGCATTCTAGTTGAACGACTGTCGC	84
D26-cy5	GCCGTAATGTGCATGTATCTCCAGGCTTTCCGCAGCGGAGCAGGAGTCCTCGGCCTTT<br>GGGCATTCTAGTTGAACGACTGTCGC**-cy5**	84
Linker	**DBCO-**TGAGCCACTGGATAC	15

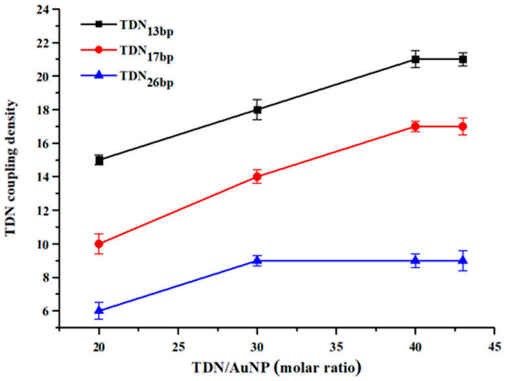

Figure S1 Optimization of the amount of TDNs
